# Damiana Used at Home

**Published:** 1904-12

**Authors:** 


### DAMIANA AS USED AT HOME.

Dr. John Uri Lloyd has made an interesting study of this drug, and *Practical Druggist* summarizes his conclusions as follows: Damiana is a Mexican shrub, its habitat being on the peninsula of Lower California, inland from La Paz. It was introduced to American medicine under a misunderstanding of its nature. It is not a Mexican drug, but a general beverage. Its qualities reside in a fragrant leaf, yielding to hot water a pleasant, harmless, tea-like beverage which, so far as history determines, has been consumed for all time by the Mexicans, and is still so employed by all classes, men, women and children alike. It is a gentle stimulant or tonic, kindly in action, pleasant to the taste, and acceptable to the stomach. Its medicinal qualities are mainly restricted, in Mexico, to cases where a gentle stimulant may be effectual, as in suppressed menses in which it is desirable to administer a hot drink in connection with a grateful aromatic that will not disturb the stomach. In other words, damiana is a homely domestic remedy, innocent of the attributes under which, in American medicine, it has for a quarter of a century, been forced to masquerade. Its medical field is now restricted, but in its true position the use of damiana may be broadened.—*Medical Times.*

